# Implementation of Interdisciplinary Province-Wide Webinar Series During the COVID-19 Pandemic by the Federation of Medical Specialists of Quebec (FMSQ): A Survey Study

**DOI:** 10.3389/fmed.2021.728715

**Published:** 2021-09-09

**Authors:** Sena Turkdogan, Tanya Chen, Tobial McHugh, Martin Tremblay, Diane Francoeur, Sam J. Daniel

**Affiliations:** ^1^Department of Otolaryngology - Head and Neck Surgery, McGill University, Montreal, QC, Canada; ^2^Department of Otolaryngology - Head and Neck Surgery, University of Toronto, Toronto, ON, Canada; ^3^Department of Otolaryngology - Head and Neck Surgery, McMaster University, Hamilton, ON, Canada; ^4^Continuing Professional Development Office, Fédération des Médecins Spécialistes du Québec, Montréal, QC, Canada; ^5^Department of Obstetrics and Gynecology, Centre Hospitalier Universitaire Sainte-Justine, Montreal, QC, Canada; ^6^Department of Pediatric Otolaryngology, Montreal Children's Hospital, Montreal, QC, Canada

**Keywords:** COVID-19, continuing professional development, medical education, virtual learning, interdisciplinary

## Abstract

**Objectives:** COVID-19 has forced a transformation in continuing professional development (CPD), shifting to virtual platforms. We report the results of a rapidly-implemented COVID-19 online interdisciplinary CPD webinar series. We aimed to determine if this virtual approach for large-scale CPD was relevant, appreciated, and effective for specialist physicians in Quebec.

**Methods and Analysis:** This was a retrospective descriptive online survey-based study. The weekly virtual educational webinars took place between March 3, 2020 to June 15, 2020, resulting in a total of 26 webinars over 16 weeks. The study included all individuals who attended any of the webinar sessions, namely specialist physicians and department chiefs. Number of participants and overall appreciation of webinar sessions were data points collected.

**Results:** Across all webinars, there were 8,500 unique specialist physicians which comprises 80.7% of the entire specialist practicing population in Quebec. Of note, every medical and surgical specialty was represented by attendance in at least one session. In total, 27,504 evaluation forms were completed out of all the sessions, meaning a 78.4% response rate. In post-webinar surveys, 97.6% of respondents agreed or strongly agreed that the webinars were pertinent to their practice and 94.6% agreed or strongly agreed that the presentation met their continuing professional needs.

**Conclusions:** This novel interdisciplinary COVID-19 webinar series is a successful and appreciated strategy to maintain CPD amidst a global pandemic. One year later, it has become a mainstay in our toolbox and we trust this unique model of large-scale interdisciplinary CPD *via* webinar sessions is useful in normal times as well as in times of crisis.

## Introduction

The novel COVID-19 pandemic and its subsequent mitigation measures have significantly disrupted medical education and professional training at multiple levels, ranging from medical student curriculum to continuing professional development (CPD) (1, 2). CPD encompasses educational activities aimed toward enhancing healthcare professionals' knowledge and skills in the provision of health care (3). Notably, interdisciplinary CPD activities have been shown to be crucial in building networking, knowledge exchange and collaboration between specialists in multidisciplinary patient care and have resulted in better quality patient care and health outcomes (4, 5). Prior to the pandemic, CPD was achieved primarily *via* in-person workshops and conferences which came to an abrupt halt due to social distancing measures, forcing many changes in the medical education sphere in order to maintain the same quality and quantity of training for medical professionals. Technology took on a significant role in adequately responding to the crisis in a safe and timely manner. Many leaders in CPD converted their speaker presentations to virtual platforms, re-organized their conferences to respect changing government regulations, and relegated networking events to a digital medium. Moreover, time was of the essence in CPD re-organization given the nature of the rapidly evolving COVID-19 crisis. As physicians were subject to continuous changes in their practice, ensuring up-to-date continuing professional development on COVID-19 became paramount in providing the safest evidence-based care to patients. Virtual education was shown to have multiple advantages during the pandemic, including its adaptability during physical distancing, its accessibility *via* asynchronous viewing, and opportunities for multi-institution participation (6, 7). For example, simulation activities in particular were shown to be extremely useful in ensuring safe medical training in response to the COVID-19 pandemic (8, 9).

In Canada, the province of Quebec quickly realized the importance of such adaptive measures as it became the epicenter for the coronavirus cases in the country. As of April 1, 2020 there were 4,611 cases in the province, constituting nearly half of the cases and nearly 30% of the country's COVID-19 mortalities (10). In wake of the impending pandemic that was impacting Quebec more heavily than the rest of Canada, the Federation of Medical Specialists of Quebec (FMSQ) needed a way to continue educating specialist physicians in the province with evidence-based COVID-related information. The FMSQ is an umbrella organization of 35 affiliated medical associations and 59 different medical specialties. With the limitations on gatherings and conferences, the FMSQ implemented a weekly interdisciplinary educational webinar series in order to reach their 10,000 members. Given the large variety of specialties encompassed by the FMSQ, an accessible dissemination medium was vital in ensuring that all associations and specialties were being reached. Constant needs assessments had to be conducted in order to keep the educational content relevant and reflective of the changing COVID-19 landscape. In this article, we report the results of a rapidly-implemented COVID-19 online interdisciplinary webinar series as a way of continuing professional development during a time of global crisis and addressing emerging educational needs. We hypothesized that this virtual approach for large-scale collaborative CPD was relevant, appreciated, and effective for specialist physicians in Quebec. We describe herein the techniques used to organize, implement, and analyze the webinar series along with physician opinions on the sessions and the self-reported impact on their practice during the COVID-19 era. Outcomes evaluated were number of participants and overall appreciation of webinar sessions.

## Materials and Methods

This study obtained ethics approval from the Research Institute of the McGill University Health Centre Research Institute and of the McGill University Health Centre Research Ethics Board.

### Patient and Public Involvement

There was no patient or public involvement in this survey as it was centered around continuing professional development of physicians in Quebec which was not relevant for the general population.

### Webinar Design

The teaching series consisted of weekly virtual educational webinars. The series took place between March 3, 2020 to June 15, 2020 and resulted in a total of 26 webinars over 16 weeks. Topics covered all 7 CanMEDS competencies: medical expert, communicator, collaborator, leader, health advocate, scholar, and professional (11). The aim of the webinars was to provide relevant educational content on COVID-19 that was pertinent for physicians from various disciplines who practiced in different settings. There were two separate webinar types: a “Specialist series” that targeted all specialist physicians of Quebec and a “Chief series” that targeted department chiefs only (Table 1). The Specialist series focused on emerging guidelines and tools pertinent to clinical practice for specialist physicians providing direct patient care as this information was constantly changing. On the other hand, the Chief series aimed to help department heads organize and manage their respective services by providing them with the necessary tools to address and adapt to their physicians' needs. Emphasis was placed on ensuring adequate patient coverage and physician safety within their departments. Despite their different goals and target populations, both webinar series were essential and complementary.

**Table 1:** Topics of webinar sessions.

**Specialist Series**	**Chief Series**
***Series targeting all specialists***	***Series targeting all department chiefs***
Pediatrics Infectious diseases Emergency medicine Intensive care Geriatrics Oncology Surgery Anesthesiology Radiology Internal medicine Public health Deontology Ethics Psychological health	Government updates Upcoming regulations Healthcare professional monitoring and reassignments Prioritization of care Resuming medical activities Provincial working group updates

**Figure 1:**
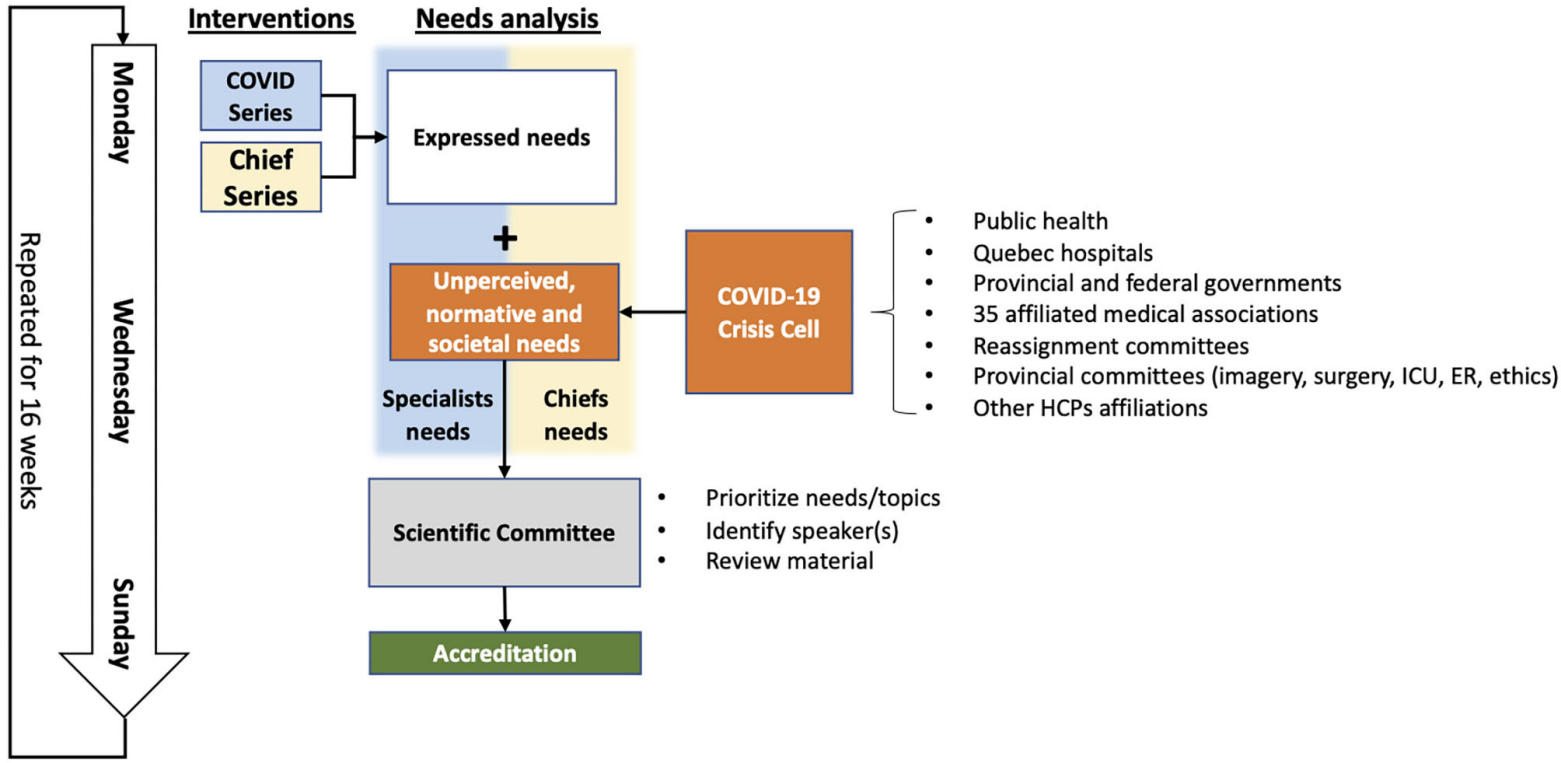
Flowchart of weekly webinar organization. ICU, intensive care unit; ER, emergency room; HCP, healthcare professional.

Figure 1 demonstrates the steps of webinar organization, including the various stakeholders involved in the needs analysis and interventions. For each session, expressed needs of participating physicians were elicited from post-webinar evaluation forms in order to determine what they wanted to learn most at the next webinar. Then, the internal FMSQ COVID-19 crisis team collaborated with individuals from the ministry health board and representatives from our affiliated medical associations to establish unperceived, normative and societal needs (see Figure 1). The scientific committee, an internal team within the FMSQ composed of members of the CPD office, then matched the physicians' expressed needs with the unperceived needs reported by the crisis cell in order to refine the learning objectives of each webinar. The experts on selected webinar topics were identified and invited as potential speakers with <5 days to prepare their presentation. Content review was then performed by independent CPD experts during Sunday evening accreditation meetings to ensure respect of administrative, ethical, and educational standards as well as relevance to target audience. Speakers were expected to be available during the accreditation meeting in the event that reviewers had questions or concerns. Presentations were also formatted and proofread by the accreditation committee in order to facilitate the speaker's role of providing high-quality content. The webinars were broadcasted on an online streaming platform and were freely accessible to all by registering in advance *via* a weekly newsletter for members or directly on the FMSQ website. All webinars were recorded and were made available on our learning management system the following day.

### Format of the Webinar

Each webinar session was scheduled to be 1 h in length, consisting of a 45-min panel of 1–4 invited speakers, followed by a 15-min discussion during which the audience could ask questions and obtain live responses from the panelists.

### Study Population

The study included all individuals who attended any of the webinar sessions organized by the FMSQ between the dates of March 3, 2020 to June 15, 2020. There were a total of 35,083 attendees across all sessions, of which 10,047 were unique. These included specialist physicians, general practitioners, and allied healthcare professionals. Eight thousand five hundred of total attendees were specialists which comprises 80.7% of the entire specialist practicing population in Quebec (8,500 specialist attendees/10,537 total specialists in Quebec) (12). In total, 27,504 evaluation forms were completed out of all the sessions, corresponding to a 78.4% response rate (27,504 evaluations completed/35,083 attendees).

### Post-Webinar Survey

All FMSQ physicians obtained an open post-webinar survey. Surveys were sent out on SurveyMonkey (San Mateo, California), an online survey platform which automatically aggregates survey answers. The survey was comprised of 18 questions distributed over two pages. Questions were selected according to the Royal College of Physicians and Surgeons of Canada CPD Activity Accreditation Standards which includes standardized questions on evaluation of individual CPD session activities (13). Participation in the survey was completely voluntary and completion of the survey was necessary in order to obtain CPD credits. During survey completion, participants had the option of pressing the back button in order to review or modify their answers. All responses were based on a four-point Likert scale: do not agree, somewhat agree, agree, strongly agree. Participants were also permitted to leave the question blank which was considered as a non-response. Participants were aware that survey responses were collected for quality improvement purposes and would only be accessible to FMSQ webinar organizers through a password-protected account, similarly to previous comparable activities. The survey was made available directly after each webinar session on the webinar platform and was also sent to each participant *via* email 1 day later which remained accessible for a duration of 2 weeks after the session in order to provide participants adequate time to reflect on the webinar.

### Collected Data

Data obtained from the webinar sessions included the date of the session, the attendance rate, participants' affiliated association or specialty, post-webinar survey responses, and any comments or questions they made on the webinar platform during the session. Concurrent COVID-19 statistics, including hospitalization rates, were collected from the provincial Quebec database: Institut national de santé publique du Québec (INSPQ). Names, email addresses, and IP addresses were also collected but were not tracked or used for identification purposes in the data analysis. Personal information was stored in a password-protected document.

### Statistical Analysis

Descriptive and quantitative analysis consisting of frequencies and percentages were used to portray characteristics of our series. Moore et al.'s outcome-based continuing medical education framework was used to assess the outcomes of educational intervention at the levels of participation and satisfaction (14).

## Results

### Moore's Level 1: Participation Data

The webinar sessions also reached a wide range of sub-specialties. Overall, there were 10,047 unique attendees as determined by unique email addresses. Table 2 demonstrates the participation data of all webinar sessions, divided into Specialist and Chief series. The majority of physicians attended more than one session and 94.7% of participants attended more than half of the total number sessions. Attendance rates by specialty were generally proportional to the size of each sub-specialty. Of note, every single one of the 35 medical associations was represented by attendance in at least one session.

**Table 2:** Participation data for Specialist and Chief sessions.

**Outcome**	**Specialist**	**Chief**	**Total (*N* =)**
Total number of participants	24,991	10,092	35,083
(average)	(1,785/webinar)	(776/webinar)	
Number of participants who attended 1 session	3,141	1,121	4,262
Number of participants who attended more than 1 session	4,286	1,499	5,785
Recording attendance	8,120	3,560	11,680
(average)	(580/webinar)	(273/webinar)	

**Figure 2:**
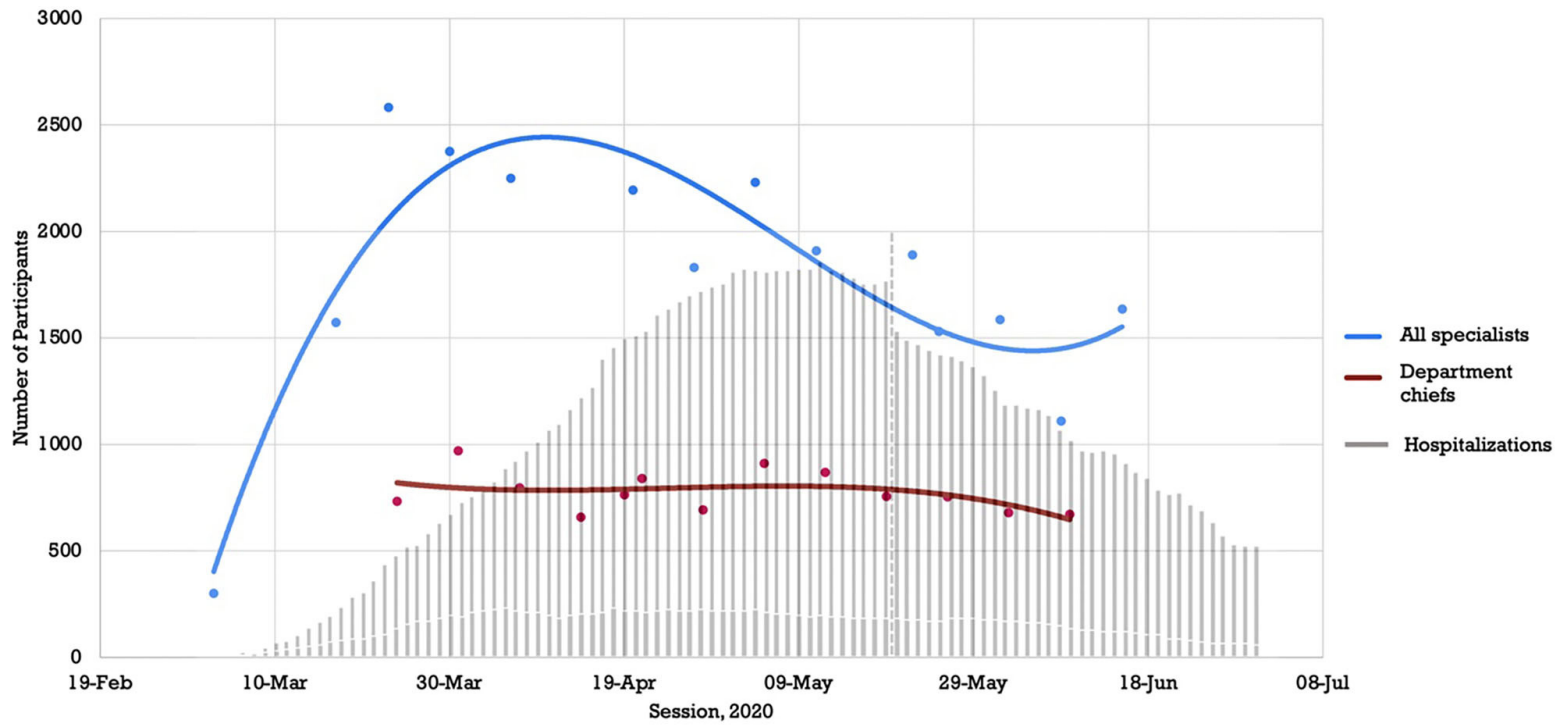
Attendance vs. Hospitalizations in Quebec over Time.

Furthermore, our analysis revealed that participation in the webinar sessions was proactive in nature rather than a delayed response to the pandemic. Figure 2 compares the timeline of the webinar attendance rates against the hospitalization rate of COVID-19 patients in Quebec using the INSPQ data. The implementation of the Specialist webinar series and the peak in attendance occurred weeks before the rise in COVID-19 hospitalizations. Attendance was also separated into Specialist sessions and Chief sessions, demonstrated, respectively, by the solid blue and red curves in Figure 2. Attendance rate amongst department chiefs remained high throughout the 16 weeks of the webinar sessions.

### Moore's Level 2: Satisfaction Data

The webinar sessions were very interactive as demonstrated by the active participation by the audience. There were 4 210 comments and questions that were individually typed and shared throughout the 26 sessions, highlighting the level of engagement amongst attendees. Of those, only 33 were voice and/or video quality-testing messages and 235 were comments related to sound quality or image quality, revealing that technical difficulties were persistently kept at a minimum.

In total, 27,504 evaluation forms were completed out of all the sessions, meaning a 78.4% response rate. Table 3 describes the results of five survey questions that were deemed the most relevant and representative of our findings. In summarizing the amalgamation of these post-webinar surveys, >95.0% of respondents agreed or strongly agreed that the webinars were pertinent to their practice and met their continuing professional needs. Between the specialist and chief sessions, there was no major difference in terms of the satisfaction. Both groups overwhelmingly agreed that the sessions were a positive experience and had a positive impact. Less than 1% of respondents elected to leave questions blank, ranging between 0.3 and 0.9% which was considered negligent and not included in final analysis.

**Table 3:** Satisfaction data for Specialist and Chief sessions.

**Outcome**	**Specialist**	**Chief**	**Total**
	(N **=, % of “Agree” or “Strongly Agree”**)		
Overall, I was satisfied with the webinar	17,108 (99.5%)	7,116 (99.2%)	24,224 (99.4%)
The webinar content was pertinent to my practice	18,934 (97.1%)	7,713 (99.3%)	26,647 (98.3%)
The speaker(s) was an expert on the webinar topic	19,101 (97.9%)	6,876 (91.7%)	25,977 (95.3%)
The speaker(s) presented material that incites me to modify certain aspects of my practice	18,652 (95.6%)	6,504 (89.7%)	25,156 (93.4%)
The webinar met my CPD needs	17,509 (97.3%)	7,319 (97.6%)	24,828 (97.4%)

## Discussion

We hereby presented a unique model of implementing continuing profession education in the reality of COVID-19. We reached 80.6% of all Quebec specialists through an interdisciplinary webinar series geared toward two distinct populations, specialist physicians and department chiefs, whose respective perceived and unperceived needs were addressed. The strongest points to these sessions were the swiftness of its development, adaptability to reflect the most current situation and efficiency of implementation which were all facilitated through the FMSQ's strong partnerships between various stakeholders in the healthcare profession. Our response rate of 78.4% for post-webinar evaluations was excellent especially when compared to other webinar-based educational studies with average response rates of ranging from 30 to 50% (15, 16). Contributors to the success of our webinars include weekly newsletters announcing the sessions, strong engagement from leaders from our affiliated medical associations, participation of established experts, reliable technology, reduction of clinical activities by participants, and cancellation of most in-person congresses. Webinars themselves demonstrated a high rate of engagement from learners as well as high participation in recordings and post-webinar surveys showed very positive feedback, whereby 97.6% of respondents agreed or strongly agreed that the webinars were pertinent to their practice. It is important to mention that it was identified as pertinent despite perhaps not being directly related to their specialty. This is especially significant given the variety amongst specialties attending sessions and the finding that every single medical specialty in Quebec was represented at least once amongst all sessions. This highlights the success of our CPD intervention and supports our execution of an agile and fluid process to stay aligned with the needs in the field. We were able to mobilize specialist physicians on interdisciplinary webinars where a variety of topics were presented and covered all CanMEDS competencies.

Over time, the slow decrease in the number of participants may be explained by the ramping up of clinical activities, increased comfort of physicians with COVID-19-related management, and increased availability of other educational media. Finally, the concept of “Zoom fatigue” was becoming well-known amongst many medical professionals leading to overall video conference meeting burnout and likely reduced attendance (17). Regardless, the importance of interdisciplinary CPD has been well-proven in literature and has been achieved through a multitude of platforms, ranging from professional speed-dating sessions to collaborative problem-based learning modules to simulation activities (4, 8, 18–20). However, few studies have discussed the CPD transformation to virtual format. Following our FMSQ initiative, many universities, affiliated medical associations and national societies have also started their own webinars, further showcasing the wide applicability of these educational sessions. Despite the challenges of transitioning CPD for the first time to virtual form amidst a constantly evolving global pandemic, participation and appreciation continued to remain high which highlight the significant value in producing these webinars. Future directions include further follow-up of impact of the webinars on actual practice and evaluation of further stages of Moore's levels of CPD assessment, such as learning and competence.

There were few limitations encountered during the implementation of the webinar series. There is a possibility of sampling bias toward physicians who were more technologically literate as the sessions took place on an online platform. To facilitate this issue, we chose a web-streaming platform where there was no need to download or install anything on the computer, but we recognize however that this still may be a factor influencing participation and attendance. Furthermore, some institutions had a firewall in place that blocked access to the web streaming platform. Lastly, though participant satisfaction was measured by post-webinar surveys, participant engagement was difficult to truly evaluate as we were only able to quantify written questions *via* online chat. Verbal questions and discussions during live sessions were not recorded, thus limiting our ability to gauge participation.

CPD has been severely affected by the COVID-19 pandemic which resulted in a rapid change in dissemination methods. This paper has described the methodology used by the FMSQ to organize, implement, and analyze a unique and efficient interdisciplinary webinar series in a timely manner allowing physicians to adjust their practice as information about COVID-19 evolved. Results from this descriptive study have demonstrated a successful strategy to quickly disseminate information and foster collaboration, transforming online knowledge transfer between multi-discipline physicians and transcending the crisis. The success of this model, its impact, and the great appreciation by our members has profoundly influenced our delivery of CPD. One year later, it has become a mainstay in our toolbox. We trust this unique model of large-scale interdisciplinary CPD *via* webinar sessions is useful in normal times as well as in times of crisis.

## Data Availability Statement

The raw data supporting the conclusions of this article will be made available by the authors, without undue reservation.

## Ethics Statement

The studies involving human participants were reviewed and approved by Research Institute of the McGill University Health Center Research Institute and of the McGill University Health Centre Research Ethics Board. Written informed consent for participation was not required for this study in accordance with the national legislation and the institutional requirements.

## Author Contributions

SD, DF, and MT conceptualized and designed the study, coordinated and supervised data collection, and critically reviewed and revised the manuscript for important intellectual content. ST, TC, and TM substantially contributed to the data collection, interpretation of data, drafted the work and revised it critically for intellectual content. All authors approved of the final version and agreed to be accountable for all aspects of the work in ensuring that questions related to the accuracy or integrity of any part of the work are appropriately investigated and resolved. The corresponding author attests that all listed authors meet authorship criteria and that no others meeting the criteria have been omitted.

## Conflict of Interest

The authors declare that the research was conducted in the absence of any commercial or financial relationships that could be construed as a potential conflict of interest.

## Publisher's Note

All claims expressed in this article are solely those of the authors and do not necessarily represent those of their affiliated organizations, or those of the publisher, the editors and the reviewers. Any product that may be evaluated in this article, or claim that may be made by its manufacturer, is not guaranteed or endorsed by the publisher.
